# Examination of the Expression Profile of Resistance Genes in Yuanjiang Common Wild Rice (*Oryza rufipogon*)

**DOI:** 10.3390/genes15070924

**Published:** 2024-07-16

**Authors:** Wang Kan, Ling Chen, Bo Wang, Li Liu, Fuyou Yin, Qiaofang Zhong, Jinlu Li, Dunyu Zhang, Suqin Xiao, Yun Zhang, Cong Jiang, Tengqiong Yu, Yunyue Wang, Zaiquan Cheng

**Affiliations:** 1 College of Plant Protection, Yunnan Agricultural University, Kunming 650224, China; mr_kanwang@163.com; 2 Biotechnology and Germplasm Resources Institute, Yunnan Academy of Agricultural Sciences/Yunnan Provincial Key Lab of Agricultural Biotechnology/Key Lab of Southwestern Crop Gene Resources and Germplasm Innovation, Ministry of Agriculture, Kunming 650205, China; cl@yaas.cn (L.C.); wangbo1040@163.com (B.W.); liuliyaas@163.com (L.L.); yinfuyou2007@126.com (F.Y.); zqf820101@aliyun.com (Q.Z.); li_jinlu@yeah.net (J.L.); zhangdunyu@163.com (D.Z.); xiaosuqin227@126.com (S.X.); zhangyun507@163.com (Y.Z.); snsdacad@163.com (C.J.); yutq76@aliyun.com (T.Y.)

**Keywords:** expression patterns, interaction network diagrams, induced expression, *Oryza*, rice blight, tissue-specific expression

## Abstract

The rice blight poses a significant threat to the rice industry, and the discovery of disease-resistant genes is a crucial strategy for its control. By exploring the rich genetic resources of Yuanjiang common wild rice (*Oryza rufipogon*) and analyzing their expression patterns, genetic resources can be provided for molecular rice breeding. The target genes’ expression patterns, subcellular localization, and interaction networks were analyzed based on the annotated disease-resistant genes on the 9th and 10th chromosomes in the rice genome database using fluorescent quantitative PCR technology and bioinformatics tools. Thirty-three disease-resistant genes were identified from the database, including 20 on the 9th and 13 on the 10th. These genes were categorized into seven subfamilies of the NLR family, such as CNL and the G subfamily of the ABC family. Four genes were not expressed under the induction of the pathogen Y8, two genes were significantly down-regulated, and the majority were up-regulated. Notably, the expression levels of nine genes belonging to the ABCG, CN, and CNL classes were significantly up-regulated, yet the expression levels varied among roots, stems, and leaves; one was significantly expressed in the roots, one in the stems, and the remaining seven were primarily highly expressed in the leaves. Two interaction network diagrams were predicted based on the seven highly expressed genes in the leaves: complex networks regulated by CNL proteins and specific networks controlled by ABCG proteins. The disease-resistant genes on the 9th chromosome are actively expressed in response to the induction of rice blight, forming a critical gene pool for the resistance of Yuanjiang common wild rice (*O. rufipogon*) to rice blight. Meanwhile, the disease-resistant genes on the 10th chromosome not only participate in resisting the rice blight pathogen but may also be involved in the defense against other stem diseases.

## 1. Introduction

As a vital food crop in China, the study of disease-resistant genes in rice and its application in ensuring food security is of far-reaching significance. Rice bacterial blight caused by *Xanthomonas oryzae pv. oryzae* is one of the main diseases in rice production in China. Therefore, the continuous exploration of excellent resistance genes to bacterial blight is the most effective way to control the disease [1]. Studies have shown that about 48 bacterial blight resistance genes have been found on 12 rice chromosomes, mainly distributed on chromosomes 4 and 11, while the related genes on chromosomes 9 and 10 have not yet been published [2]. It is worth noting that nearly 85% of these bacterial blight resistance genes are derived from cultivated rice, compared with relatively few resistance genes found in wild rice [3]. Common wild rice (*O. rufipogon*) has rich genetic diversity, including many beneficial genes, such as those for high yields, disease resistance, and stress tolerance. The genetic relationship between common wild rice and cultivated rice is the closest; both AA genomes and the disease-resistant genes contained in them are more easily applied to cultivated rice [4]. As one of the origins of Asian cultivated rice, Yuanjiang common wild rice is the common wild rice with the highest altitude (780 m) in China so far [5]. It has its unique geographical advantages and has attracted the attention of scholars. At present, some specific genes have been cloned from Yuanjiang common wild rice, such as the control of seed shattering gene *SHA1*, loose panicle trait gene *OsLG1*, long spine awn gene *LABA1*, creeping growth trait gene *PROG1* and bacterial blight resistance gene *Xa47* [6,7,8,9,10,11]. Therefore, it is particularly important to understand the molecular mechanism of disease resistance in Yuanjiang common wild rice and to explore candidate genes for improving rice resistance.

Among the resistance genes, NLR genes are widely distributed in the genomes of animals and plants and are one of the largest gene families. Current studies have found that NLR genes are also significant in plant growth and signal transduction. In addition, when plants are subjected to abiotic and biotic stresses, the expression of NLR genes is up-regulated to enhance plant resistance [12]. The protein encoded by the complete NLR gene has a nucleotide binding site (NBS) and a leucine-rich repeats (LRR) domain. However, some NLR proteins have other domains at the N-terminus, such as multiple NBS domains, or lack the LRR domain at the C-terminus. According to the different domains, these gene families can be divided into the following categories: NBS (N), NBS-LRR (NL), CC-NBS (CN), CC-NBS-LRR (CNL), TIR-NBS (TN), TIR-NBS-LRR (TNL), CC-TIR-NBS (CTN), RPW8-NBS (PN), RPW8-CC-NBS (PCN), and RPW8-NBS-LRR (PNL) [13]. Among them, the RPW8 domain is related to the broad-spectrum resistance of powdery mildew pathogens [14]. The TIR domain plays an important role in pathogen detection, while the CC domain is related to protein–protein interaction [15]. The NBS domain is highly conserved, binds to ATP or GTP, regulates protein activity, and plays a major role in signal transduction and resistance specificity. The LRR domain interacts with proteins. LRR is involved in the recognition and signal transduction of pathogen avirulent proteins, which is the main reason for the specific recognition of pathogen races by resistance genes [16]. With the development of high-throughput sequencing, researchers have performed genome-wide identification and classification of NLR gene families in rice [17]. Currently, 623–725 NLR genes have been found in the rice genome [18]. These genes confer resistance to rice blast, bacterial blight, rice planthopper, and other pests and tolerance to cold damage. In terms of rice blast, most of the cloned rice blast resistance genes are NLR genes, such as *Pi63*, *Pi1*, *Pi2*, *Pi5*, *Pia*, and so on [19,20,21,22,23]. The known NLR resistance genes are only *Xa1* and its four alleles (*Xa2*, *Xa31*, *Xa14*, and *Xa45*) [24], as well as *Xa47* identified by our laboratory [10,11]. Most of the known rice planthopper resistance genes are also NLR genes, such as *Bph14*, *Bph26*, *Bph1* and *Bph2*, *Bph7*, *Bph9*, *Bph10*, *Bph18*, *Bph21* and *Bph26* allelic, encoding the CC-NBS-LRR protein [25,26]. The cold tolerance gene *OsPi304* also encodes the CC-NBS-LRR protein [27].

ATP-binding cassette transporters (ABC transporters) are a class of classical transmembrane transporters with diverse functions. They are widely found in various organisms and are one of the largest protein families in existing organisms [28]. They can use the energy produced by ATP hydrolysis to transport the substrate into or outside of the cell. Many kinds of substrates include drugs, toxins, nutrients, and metabolites. The diversity of substrates promotes ABC transporters to participate in various biological processes such as hormone transport, signal transduction, cell detoxification, disease defense, and antigen transmission [29]. For plants, ABC transporters play an important role in regulating plants’ adaptability and disease resistance to the external environment [30]. The ABC family genes include 10 subfamilies such as ABCA~ABCJ. To date, 128 ABC members have been identified in the rice genome, including 6 ABCA members, 27 ABCB members, 17 ABCC members, 3 ABCD members, 2 ABCE members, 6 ABCF members, 50 ABCG members, and 17 ABCI members [31]. However, the functions of most of these members have not been reported. At present, only the gene *OsABCB14*, related to auxin input and iron balance [32], the gene *OsABCB23*, maintaining plant iron balance [33], the gene *OsPDR20*, involved in cadmium and zinc accumulation and detoxification in rice [34], the gene *OsABCC13*, involved in the transport of phytic acid and the accumulation of phosphorus-containing products in rice seeds [35], the gene *OsABC25*, alleviating aluminum toxicity in rice plants [36], and the gene *OsABCC17*, involved in the regulatory network of rice chloroplast development, have been reported [37]. The ABC members related to disease resistance in rice have not been reported so far.

In view of the current scarcity of bacterial blight resistance genes on rice chromosomes 9 and 10, as well as the lack of NLR and ABC bacterial blight resistance genes, in this study, through the in-depth analysis of the Rice Genome Annotation Project database, several NLR family genes and ABC transporter family genes located on rice chromosomes 9 and 10 were targeted for in-depth exploration. These genes’ induced expression and tissue-specific expression were analyzed by fluorescence quantitative PCR. The excellent genes related to bacterial blight resistance were screened from the genome of Yuanjiang common wild rice, which provided valuable genetic resources for rice disease resistance breeding in China.

## 2. Materials and Methods

### 2.1. Materials

Plant material: Yuanjiang common wild rice is a perennial herb in the genus Gramineae. It is collected from the Yuanjiang Area of Yunnan Province, China, at an altitude of 780 m. The plant was planted in the greenhouse of the Institute of Biotechnology and Germplasm Resources, Yunnan Academy of Agricultural Sciences.

Bacterial blight strain: In this study, Yunnan epidemic strain Y8, non-pathogenic to Yuanjiang common wild rice, was selected as the test strain.

### 2.2. Methods

#### 2.2.1. Gene Download

Genes were downloaded from the Rice Genome Annotation Project database (http://rice.uga.edu/index.shtml (accessed on 20 January 2024)) using the Landmark or Region function of the Rice Genome Browser tool. The chromosome number was entered in its toolbar, and then the resistance genes published on rice chromosome 9 and chromosome 10 were downloaded.

#### 2.2.2. Pathogen Culture

Before inoculation, the strain was inoculated on NA medium and cultured at 28 °C ± 2 °C for 48~72 h. During the inoculation process, sterile distilled water was used to elute the strain, which was uniformly suspended and prepared into a concentration of 3 × 108 cfu/mL (OD_600_ = 0.5). The NA medium formula is as follows: beef extract 3 g, yeast extract 1 g, peptone 5 g, sucrose 10 g, agar 17 g, add water to 1000 mL, and adjust the PH to 6.8~7.0.

#### 2.2.3. Inoculation Induction Treatment

Three strains of Yuanjiang common wild rice in the same growth environment were inoculated, and the second leaves were cut at 8 time points of 0 h, 12 h, 24 h, 36 h, 48 h, 60 h, 72 h, and 84 h, respectively. Quickly freeze rice materials with liquid nitrogen and store them in a −80 °C ultra-low-temperature refrigerator for future experimental use.

#### 2.2.4. Fluorescence Quantitative PCR

Real-time PCR Total RNA was extracted using the Eastep Super RNA LS1040 kit (Promega, Madison, WA, USA). The RNA was reverse transcribed into cDNA using the HiScript III RT SuperMix for the qPCR kit (Vazyme Biotech Co., Ltd., Hongfeng Science and Technology Park, Kechuang Road, Nanjing Economic and Technological Development Zone, Jiangsu Province, China). According to the CDS sequence of the NBS-LRR resistance gene, the gene expression primers were designed using the NCBI online primer design tool (https://www.ncbi.nlm.nih.gov/tools/primer-blast/index.cgi?LINK_LOC=BlastHome (accessed on 21 January 2024)). The specific amplification primer information is shown in Table S1. The *Actin* gene [38] was used as the internal reference gene, and the ratio of the reaction system was carried out concerning ChamQ Univer-sal SYBR qPCR Master Mix (Vazyme Biotech Co., Ltd., Hongfeng Science and Technology Park, Kechuang Road, Nanjing Economic and Technological Development Zone, Jiangsu Province, China). The two-step method was used for fluorescence quantitative PCR amplification. The amplification procedure was as follows: pre-denaturation at 95 °C for 60 s; denaturation at 95 °C for 10 s, annealing at 60~63 °C for 10 s, 40 cycles. The relative expression of the gene was calculated using the 2^−ΔΔCt^ method [39], and the data were collated, analyzed, and plotted using Excel.

#### 2.2.5. Bioinformatics Analysis

The String website (https://string-db.org/ (accessed on 22 January 2024)) was used to predict the interaction network between the tested proteins and annotate their functions. On this basis, the interaction data between proteins were visualized by GraphPad Prism 8.0.1 software. DNAMAN6.0 software was used to perform multiple sequence alignments of the amino acid sequences of the tested proteins to analyze the similarity of amino acid sequences. WoLF PSORT (https://wolfpsort.hgc.jp/ (accessed on 22 January 2024)) and CELLO v.2.5 (http://cello.life.nctu.edu.tw/ (accessed on 22 January 2024)) were used to predict the subcellular localization of the tested proteins.

## 3. Results

### 3.1. Basic Information of NLR and ABC Family Genes in Rice Chromosomes 9 and 10

**Figure 1:**
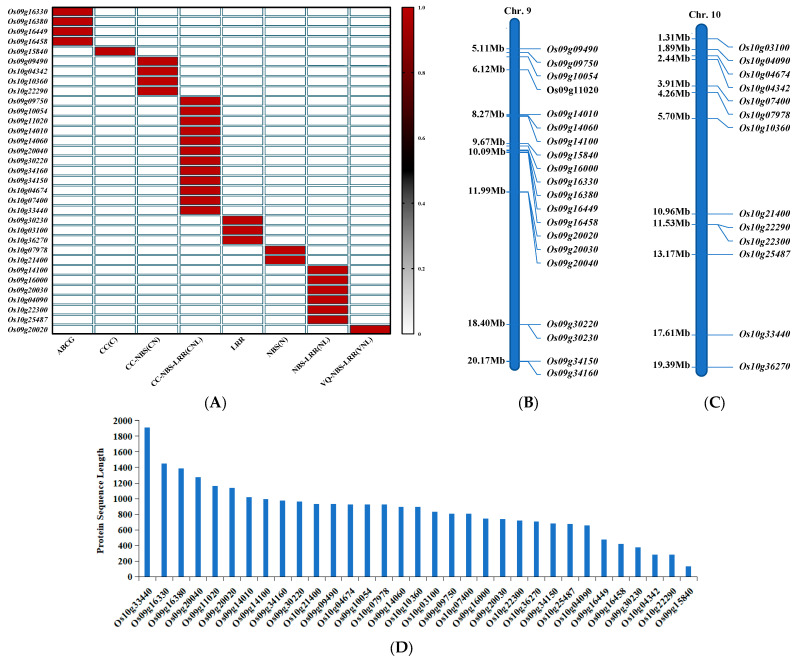
NLR and ABC family genes downloaded from rice chromosomes 9 and 10. Classification of 33 key genes (**A**). Distribution of genes on chromosomes 9 and 10 (**B**). Protein sequence length of 33 key genes (**C**). 33 protein sequence lengths (**D**).

In the Rice Genome Annotation Project database, the disease resistance genes annotated on rice chromosomes 9 and 10 were downloaded. These genes include multiple NLR genes and ABC gene family members. After screening, 33 key genes were identified, of which 29 belonged to the NLR family, the NLR family includes CC (C), CC-NBS (CN), CC-NBS-LRR (CNL), LRR, NBS (N), NBS-LRR (NL), VQ-NBS-LRR (VNL) (Figure 1A), and the other 4 belonged to the ABCG subfamily. CC-NBS-LRR (CNL)-type genes account for a large proportion of the NLR family, about 41.38%. The proportion of LRR is about 10.35%, the proportion of NBS-LRR (NL) is about 20.7%, and the proportion of NBS (N) is about 6.9%. In contrast, CC (C) and VQ-NBS-LRR (VNL) types have relatively few genes, with only one instance per type (Figure 1A). Specific to the chromosome, there are 16 NLR genes and four ABCG genes on chromosome 9, while all NLR genes are on chromosome 10, a total of 13. The distribution patterns of these genes on chromosomes show a certain degree of aggregation (Figure 1B,C), but the proteins they encode are significantly different in size (Figure 1D), indicating that these genes do not exist in the form of repeated mapping on chromosomes, but are distributed as single-copy genes.

### 3.2. The Expression of 33 Downloaded Genes Induced by Pathogenic Bacteria

#### 3.2.1. Expression Pattern Analysis of ABCG Subfamily Genes

**Figure 2:**
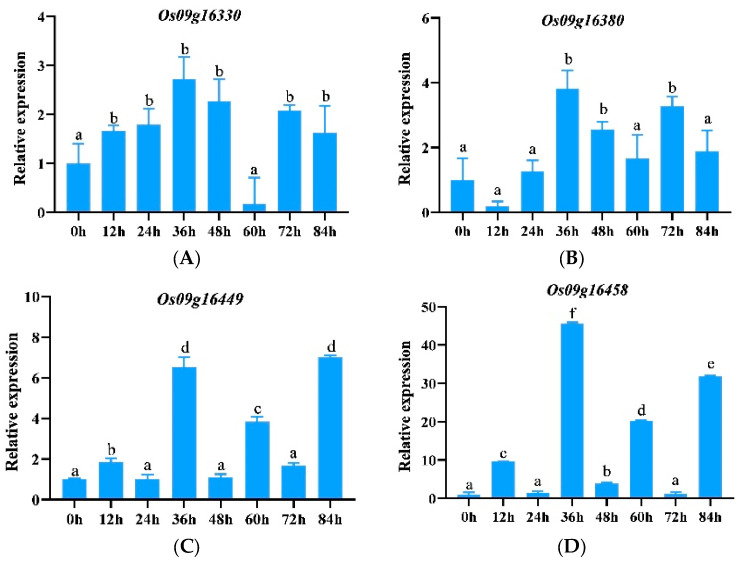
Expression of ABCG subfamily genes in response to the pathogen Y8. (**A**–**D**) represent genes *Os09g16330*, *Os09g16380*, *Os09g16449*, and *Os09g16458*.

Among the 33 genes downloaded, *Os09g16330*, *Os09g16380*, *Os09g16449*, and *Os09g16458* belong to the ABCG family. Fluorescence quantitative PCR was used to further analyze the expression patterns of these genes induced by Y8. The results showed that these four genes were up-regulated after infection with Y8, especially at 36 h after infection with Y8 (Figure 2). It can be seen that although the expression levels of *Os09g16330* and *Os09g16380* increased, they were relatively low, 2.72 times and 3.82 times that of the initial expression levels, respectively (Figure 2A,B). In contrast, the expression levels of *Os09g16449* and *Os09g16458* significantly increased, 6.52 times and 45.57 times that of 0 h, respectively (Figure 2C,D).

#### 3.2.2. Analysis of Expression Patterns of NLR Family Genes of the CC (C) Type and CC-NBS (CN) Type in this Study

**Figure 3:**
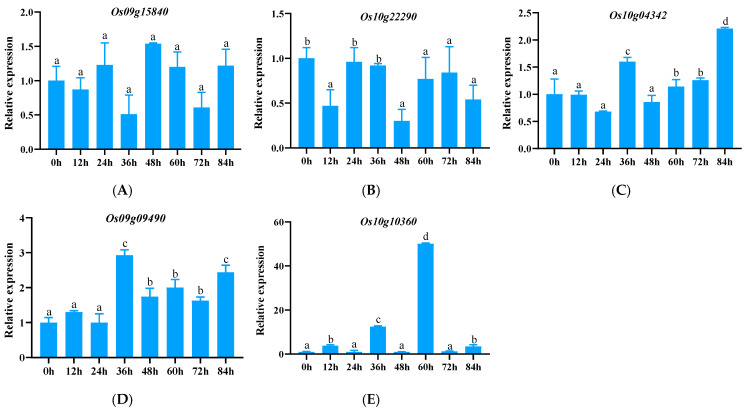
Expression of CC-type and CC-NBS-type NLR family genes in response to the pathogen Y8. (**A**–**E**) represent genes *Os09g15840*, *Os10g22290*, *Os10g04342*, *Os09g09490*, and *Os10g10360*.

It was found that C-type genes included *Os09g15840*, and CC-NBS-type genes included *Os09g09490*, *Os10g04342*, *Os10g10360*, and *Os10g22290*. The expression patterns of these genes were analyzed by fluorescence quantitative PCR; it was found that the expression level of the C-type *Os09g15840* gene remained relatively stable from 0 h to 84 h. This suggests that it may not be involved in the immediate defense response to specific pathogens (Figure 3A). It should be noted that CN-type genes significantly responded to the stress of *Xoo*. Specifically, the expression of the *Os10g22290* gene was significantly down-regulated after encountering pathogens. This gene showed a significant decrease in expression at different time points after inoculation (Figure 3B). In sharp contrast, *Os09g09490*, *Os10g04342*, and *Os10g10360* expression levels were up-regulated under pathogen induction. Among them, *Os09g09490* and *Os10g04342* were low expression genes (Figure 3C–E), and their highest expression levels were only 2.21 to 2.93 times the initial expression level. *Os10g10360* is a highly expressed gene, and its expression level peaks at 60 h, 50.04 times the initial expression. Especially at 12 h, 36 h, and 84 h, the expression level remained more than three times the initial expression level (Figure 3E).

#### 3.2.3. Expression Pattern Analysis of CC-NBS-LRR (CNL)-Type NLR Family Genes

**Figure 4:**
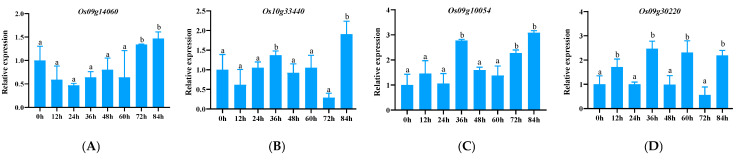

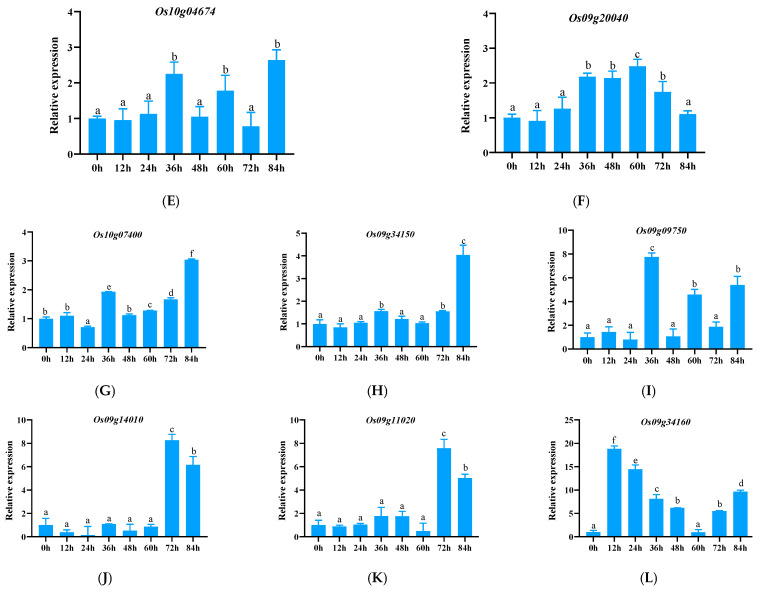
Expression of CC-NBS-LRR-type NLR family genes in response to the pathogen Y8. (**A**–**L**) represent genes *Os09g14060*, *Os10g33440*, *Os09g10054*, *Os09g30220*, *Os10g04674*, *Os09g20040*, *Os10g07400*, *Os09g34150*, *Os09g09750*, *Os09g14010*, *Os09g11020*, and *Os09g34160*.

CNL genes were up-regulated by pathogens. Among them, the expression levels of *Os09g14060* (Figure 4A), *Os10g33440* (Figure 4B), *Os09g10054* (Figure 4C), *Os09g30220* (Figure 4D), and *Os10g04674* (Figure 4E) at most time points were not significantly different from the initial time point (0 h), suggesting that their potential role in plant disease resistance may not be significant. On the other hand, although *Os09g20040* and *Os10g07400* genes showed a significant upward trend, their expression levels were still at a low level (Figure 4F,G). More interestingly, for the four genes *Os09g34150* (Figure 4H), *Os09g09750* (Figure 4I), *Os09g14010* (Figure 4J), and *Os09g11020* (Figure 4K), although their up-regulated expression trends are not obvious, their expression levels are significantly increased at certain time points, showing a specific expression pattern. Of particular note is the *Os09g34160* gene, which showed significant early up-regulated expression in the CC-NBS-LRR family and maintained a high expression level at most time points (Figure 4L). The average relative expression fluctuated between 5.52 and 18.83. Only at 60 h, the expression level did not show a significant difference compared with the initial time point, which may be because the plant has entered a new physiological state at this time point or the stress of the pathogen has been alleviated.

#### 3.2.4. Expression Pattern Analysis of Other NLR Family Genes

**Figure 5:**
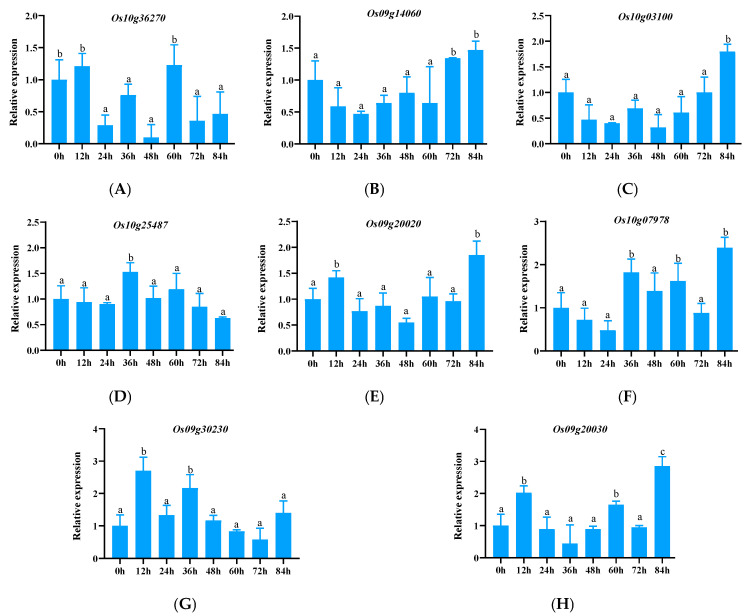
Expression of NLR family genes with non-CC domains in response to the pathogen Y8. (**A**–**H**) represent genes *Os10g36270*, *Os09g14060*, *Os10g03100*, *Os10g25487*, *Os09g20020*, *Os10g07978*, *Os09g30230*, and *Os09g20030*.

The remaining NLR family genes include LRR-type genes (*Os09g30230*, *Os10g03100*, and *Os10g36270*), and only contain NBS (N)-type genes (*Os10g07978* and *Os10g21400*), NBS-LRR (NL)-type genes (*Os09g14100*, *Os09g16000*, *Os09g20030*, *Os10g04090*, *Os10g22300*, and *Os10g25487*), and VQ-NBS-LRR (VNL) type gene *Os09g20020*. Among these, four genes, *Os10g21400*, *Os09g14100*, *Os10g04090*, and *Os10g22300*, were not expressed during the whole period of Y8 induction, and the relative expression level was 0. The expression of the other eight genes is shown in Figure 5. It can be found from Figure 5 that the expression level of the *Os10g36270* gene was significantly decreased. Although the expression of the remaining seven genes was up-regulated, their relative expression levels were relatively low and were not active at most time points, indicating that these four genes were low-level expression genes and may not play a leading role in the disease resistance of Yuanjiang common wild rice.

#### 3.2.5. Tissue-Specific Expression Analysis of Significantly Up-Regulated Genes

**Figure 6:**
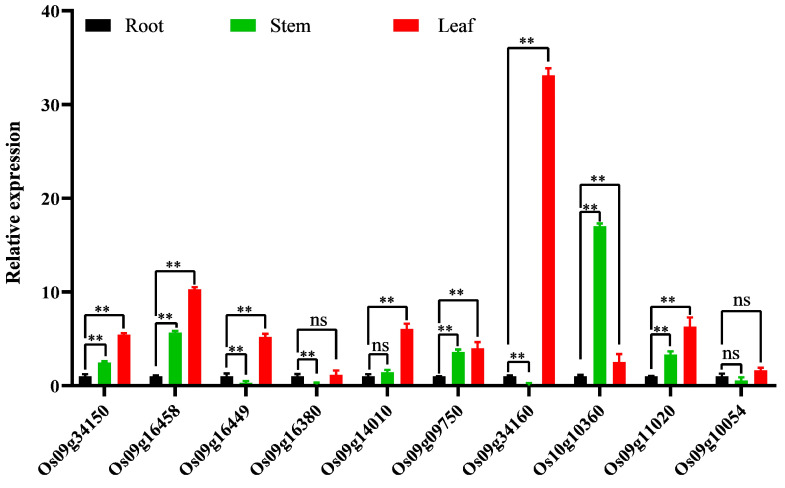
Tissue-specific expression of the top 10 genes highly induced by the pathogen Y8. The data are presented as the mean χ ± SE (*n* = 3) of three biological replicates. Analysis of variance was conducted using Bonferroni’s multiple comparisons test. ** means significant differences at the 0.05 level. ^ns^ means no significant differences.

According to the above 33 genes induced by pathogen expression results, 9 significantly up-regulated genes (*Os09g09750*, *Os09g11020*, *Os09g14010*, *Os09g16458*, *Os09g16449*, *Os09g16380*, *Os09g34160*, *Os09g34150*, and *Os10g10360*) were selected for tissue-specific expression analysis. At the same time, the gene *Os09g100054*, which is basically not expressed, was selected as the control. As shown in Figure 6, the control gene *Os09g100054* was not differentially expressed in roots, stems, and leaves. The ABCG gene *Os10g16380* was mainly expressed in roots, and the CC-NBS gene *Os10g10360* was mainly expressed in stems. The remaining seven genes were expressed in multiple tissues. Among them, the ABCG gene *Os09g16458* was significantly expressed in stems and leaves, while *Os09g16449* was significantly expressed in roots and leaves. Among the CC-NBS-LRR genes, Os09g34150, Os09g14010, *Os09g09750*, and *Os09g11020* were significantly expressed in stems and leaves, and *Os09g34160* was significantly expressed in roots and leaves. These seven genes were highly expressed in leaves, especially the *Os09g34160* gene, whose average relative expression level reached 33.13.

#### 3.2.6. Prediction of ABCG-Type Regulatory Network of Highly Expressed Genes

**Figure 7:**
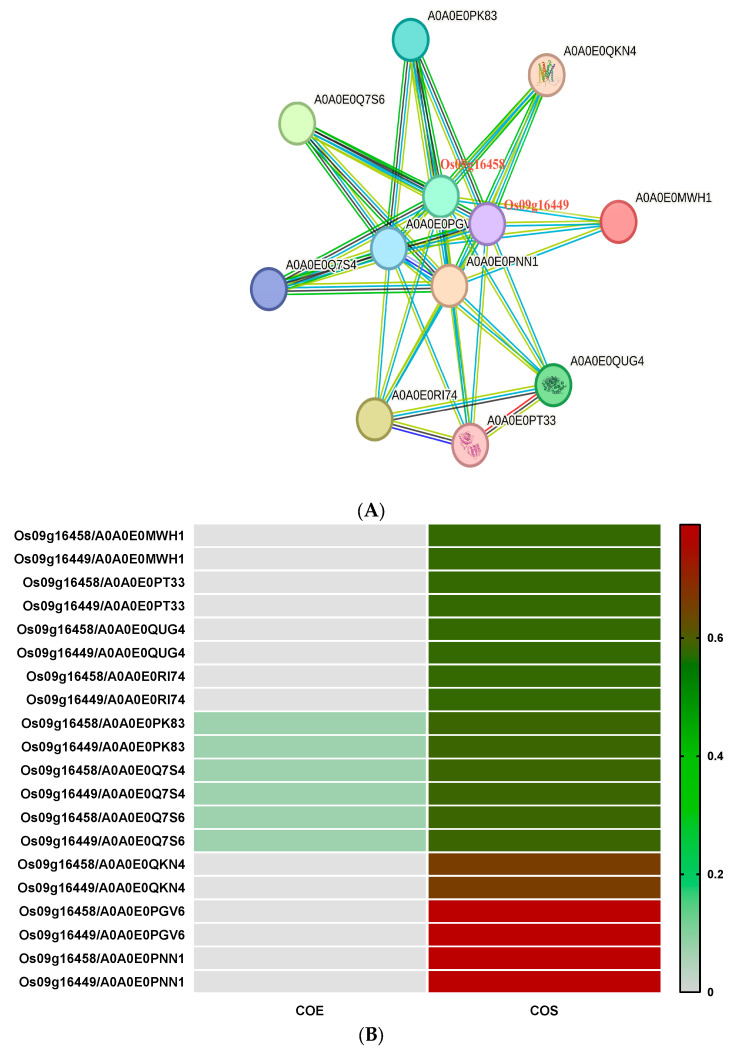
Construction and analysis of the ABCG protein regulatory network. Protein interactions mediated by ABCG-type proteins Os09g16458 and Os09g16449 (**A**). Interaction information between Os09g16458 and Os09g16449 with ten predicted proteins (**B**). COE: Co-expression. COS: Score of protein interactions predicted based on amino acid domain structures.

After pathogen stimulation, *Os09g16458* and *Os09g16449*, two ABCG genes, showed significantly high levels of expression, and their expression levels were particularly prominent in leaves. By using the regulatory network constructed by the online software String (https://cn.string-db.org/ (accessed on 8 February 2024)) (Figure 7), we could predict the protein functions in the network (Table 1). It was found that *Os09g16458* and *Os09g16449* did not interact directly but indirectly formed a regulatory network through direct interaction with 10 proteins, including AOAOEOPK83. In this network, there are three proteins, A0A0E0PK83, A0A0E0Q7S4, and A0A0E0Q7S6, whose functions have not been revealed. They are mainly co-expressed with *Os09g16458* and *Os09g16449* in the nucleus or plasma membrane. In addition, the two E3 binding enzyme proteins, A0A0E0PGV6 and A0A0E0PNN1, located in the nucleus had the highest degree of interaction with *Os09g16458* and *Os09g16449*. Most other proteins were mainly distributed in chloroplasts, and the interaction intensity with *Os09g16458* and *Os09g16449* was the same. Of these proteins, only the biological function of the glyphosate herbicide resistance protein A0A0E0PT33 is known, and the remaining protein functions are still unknown.

#### 3.2.7. Prediction of CC-NBS-LRR (CNL)-Type Regulatory Network of Highly Expressed Genes

**Figure 8:**
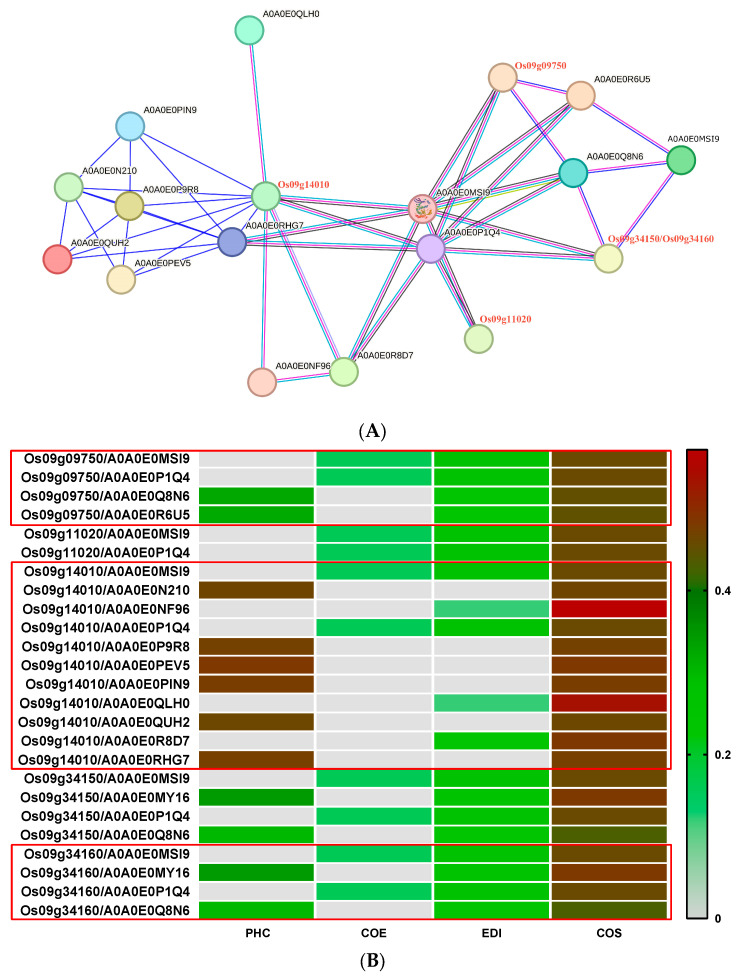
Construction and analysis of the CNL protein regulatory network. Protein interactions mediated by CNL-type (**A**). Interaction information between CNL with ten predicted proteins (**B**). PHC: Phylogenetic co-occurrence. COE: Co-expression. EDI: Experimentally determined interaction. COS: Score of protein interactions predicted based on amino acid domain structures.

**Figure 9:**
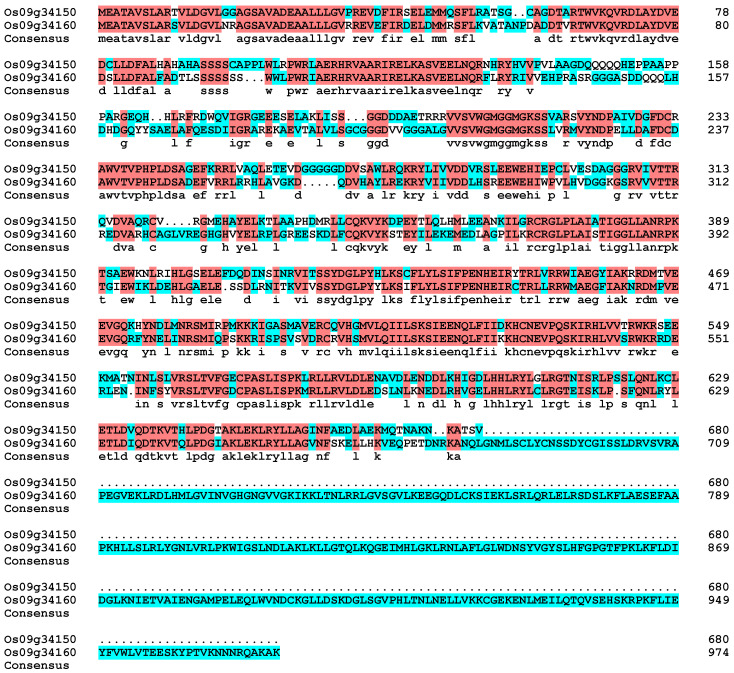
Amino acid sequence comparison of *Os09g34150* and *Os09g34160.* Blue and white are differential amino acids; red is the same amino acid.

The CNL high-expression genes include *Os09g34150*, *Os09g14010*, *Os09g09750*, *Os09g11020*, and *Os09g34160*. We used the online software String (https://cn.string-db.org/ accessed on 10 February 2024) to construct the regulatory network of these genes (Figure 8A), and predicted the function of the proteins in the network (Table 2). In the regulatory network, *Os09g34150* and *Os09g34160* are regarded as the same gene because they interact with the same protein, and the corresponding prediction scores are exactly the same (Figure 8B). However, online prediction results showed that *Os09g34150* was localized in the cytoplasm, while *Os09g34160* may be a nuclear-cytoplasmic double-localization protein (Table 2). Amino acid sequence alignment showed significant differences in amino acid sequences between *Os09g34150* and *Os09g34160* (Figure 9), proving that the two genes were not the same.

Figure 8 shows that the five highly expressed CNL genes do not directly interact with each other but interact with some of the 14 proteins to build a complex regulatory network. In this network, two protein kinase subfamily genes, A0A0E0MSI9 and A0A0E0P1Q4, mainly located in the cytoplasm, play a bridge role and closely link these five CNL genes. In particular, A0A0E0MSI9 and A0A0E0P1Q4 interact directly with and co-express with five CNL proteins, respectively. In particular, the CNL protein *Os09g11020*, mainly located in the cytoplasm, only directly interacts with A0A0E0MSI9 and A0A0E0P1Q4 and indirectly interacts with other proteins. In addition, proteins A0A0E0MY16 and A0A0E0Q8N6 directly interact with *Os09g34150* and *Os09g34160*. Although both *Os09g09750* and *Os09g14010* are located in the nucleus, the proteins interacting with them are different. There were only four proteins interacting with *Os09g09750*, including A0A0E0MSI9 and A0A0E0P1Q4, A0A0E0Q8N6, and A0A0E0R6U5. In addition to the aforementioned A0A0E0MSI9 and A0A0E0P1Q4 proteins, nine proteins, such as A0A0E0N210, directly interact with *Os09g14010*. Among these nine proteins, A0A0E0NF96, A0A0E0QLH0, and A0A0E0R8D7 can not only be predicted by the domain but can also be confirmed by related experiments that they interact with *Os09g14010*.

In this study, we identified 14 proteins that interact with 5 CNL proteins, which are widely distributed in cells, including the cytoplasm, nucleus, plasma membrane, and extracellular vesicles. It is worth noting that only A0A0E0NF96 is specifically localized to extracellular vesicles and interacts with avirulent proteins secreted by pathogenic bacteria. In terms of plasma membrane localization proteins, we found three proteins: A0A0E0QUH2, A0A0E0PIN9, and A0A0E0P9R8. Although their specific functions are unclear, their localization suggests that they may play an important role in the dynamic changes in cell membranes and signal transduction. In the nuclear localization protein, A0A0E0QLH0 and A0A0E0MY16 contain two key domains, RING and PWWP. These two domains have a wide range of effects in regulating the cell cycle, DNA repair, and gene expression. The data in Table 1 also suggest that A0A0E0PEV5 may be located in both the nucleus and the cytoplasm, but the specific function of this co-localization protein remains to be explored. The types of cytoplasmic localization proteins are the most abundant, including eight protein sites such as A0A0E0MSI9. These sites not only contain a variety of important functional proteins, such as protein kinase subfamily genes A0A0E0MSI9 and A0A0E0P1Q4, F-box domain protein A0A0E0N210, and NLR proteins, they play a vital role in biological processes such as cell signal transduction and immune response.

## 4. Discussion

### 4.1. CC-NBS-LRR (CNL) Family Genes Play a Key Role in the Resistance of Yuanjiang Common Wild Rice to Bacterial Blight

The research results show that the CC-NBS-LRR gene is the main component in monocotyledonous plants, while both CC-NBS-LRR and TIR-NBS-LRR genes exist in dicotyledonous plants [40]. In this study, we selected 33 genes from rice chromosomes 9 and 10, of which 12 were CC-NBS-LRR genes. In addition, among the ten genes highly expressed by pathogen induction, except for three genes with ABC transport function and one CC-NBS gene on chromosome 10, the rest are CC-NBS-LRR genes. This finding indicates that the CC-NBS-LRR gene plays a crucial role in the immune defense of rice, especially the CC domain. Studies have shown that the CC structure is a key functional domain of many proteins, including structural proteins, kinesin, and transcription factors. About 5% of known proteins contain a CC structure [41]. Related studies have confirmed that the oligomerization of the CC domain is a key step in NLR activation, which is closely related to downstream immune signal transduction. If this region is missing, the remaining NBS-LRR cannot trigger the immune response of plants. The CC structure alone can trigger an appropriate immune response [42]. Therefore, discovering these genes is essential for understanding the operation of the plant immune system.

In the study of CNL genes, the expression levels of *Os09g34150*, *Os09g14010*, *Os09g09750*, *Os09g11020*, and *Os09g34160* were not only significantly increased under the induction of *Xoo*, but also particularly active in leaves. Given that bacterial blight mainly affects leaves, the high expression of these genes in leaves may suggest their key role in resisting this leaf disease. Therefore, it is reasonable to speculate that these five genes play a crucial role in the resistance of Yuanjiang common wild rice to bacterial blight. In addition, the *Os10g10360* gene can also be induced by *Xoo*, and its expression level is much higher than that of other types of genes. It is worth noting that this gene is mainly expressed in stems rather than leaves, suggesting that it may have special functions in resisting stem pests and diseases, such as sheath blight, rice blast, rice bacterial basal rot, rice bakanae disease, rice sheath rot, and rice planthopper [43]. More interestingly, the *Os10g10360* gene is the only gene on chromosome 10 that is highly induced by pathogenic bacteria, which further highlights its possible significant role in rice disease resistance and stress resistance. The function and potential of these genes need to be further explored and studied.

### 4.2. Five CC-NBS-LRR (CNL) Proteins, including Os09g34150, Mediate Complex Regulatory Pathways

The immune system of plants is like a delicate triangular structure, the first feature of which is immune recognition, the second is signal transduction, and, finally, the implementation of defense. In this study, five CLR proteins, Os09g34150, Os09g14010, Os09g09750, Os09g11020, and Os09g34160 [44], played a crucial role in the battle against *Xoo* in Yuanjiang common wild rice. To this end, the five protein-mediated disease resistance signal regulatory networks were constructed with the help of bioinformatics. At the level of immune recognition, plants have evolved NLR proteins, which, like sharp scouts, can directly recognize the effectors secreted by pathogens or recognize the modification of host proteins caused by effectors, thus triggering defense responses [45]. This means that some or all of the five CLR proteins in this study have identified the effector proteins secreted by *Xoo* and activated the disease resistance signal. Signal transduction involves multiple levels, such as membrane structure, a functional signal system, and intracellular signal system activation [46]. The plasma membrane localization proteins A0A0E0QUH2, A0A0E0PIN9, and A0A0E0P9R8 found in this study may be important proteins in the functional signal system of membrane structure; the eight proteins, A0A0E0MSI9, A0A0E0N210, A0A0E0R8D7, A0A0E0RHG7, A0A0E0P1Q4, A0A0E0Q8N6, A0A0E0R6U5, A0A0E0PEV5, and the protein A0A0E0NF96, located in extracellular vesicles, may be involved in the activation of intracellular signaling systems. As for the defense response, the defense proteins in the nucleus respond to the disease resistance signal and transfer the disease resistance signal to the cytoplasm to play a disease-resistant role [47]. Therefore, it is speculated that this study found that the two proteins A0A0E0QLH0 and A0A0E0MY16 located in the nucleus responded to the disease resistance signal and transferred the signal into the cytoplasm through the nucleoplasmic localization protein A0A0E0PEV5 to play a disease resistance role. In general, the five CNL proteins identified in this study played an important role in mediating the disease resistance of Yuanjiang common wild rice, and the predicted fourteen interacting proteins were involved in the whole disease resistance pathway mediated by these five proteins, but the specific conclusions need further experimental verification. This study is expected to systematically analyze the mechanism of broad-spectrum high resistance to bacterial blight in Yuanjiang common wild rice and provide strong theoretical support for future agricultural development.

### 4.3. ABCG-like Proteins Are Essential in the Disease Resistance Process of Yuanjiang Common Wild Rice

Os09g16458 and Os09g16449, two ABCG proteins, not only showed significant high expression under the stimulation of pathogens but also showed abnormal expression levels in leaves. Their expression patterns are similar to those of CNL proteins in previous studies. This finding profoundly reveals that Os09g16458 and Os09g16449 play an important role in the disease resistance mechanism of Yuanjiang’s common wild rice. However, it is worth noting that the regulatory networks mediated by these two proteins are significantly different from those mediated by CNL proteins. Studies have revealed that ABC transporters in plants play a vital role in the transport of exogenous substances and secondary metabolites, and they play an indispensable role in the detoxification of plants and the battle against pathogen invasion [48]. In the regulatory network predicted in this study, there is a striking phenomenon: glyphosate herbicide resistance protein A0A0E0PT33 even appeared in this network. It is speculated that Os09g16458 and Os09g16449 may also play a key role in the herbicide resistance of Yuanjiang’s common wild rice. Although the function of most proteins in this regulatory network is still a mystery, the involvement of Os09g16458 and Os09g16449 is almost certain. Therefore, it can be concluded that Os09g16458 and Os09g16449 are essential for the disease-resistant process of Yuanjiang common wild rice. However, the conclusion of all this must be further confirmed by subsequent functional verification experiments.

## 5. Conclusions

This paper successfully identified and screened 33 important resistance genes on chromosomes 9 and 10 from the rice genome annotation database. These genes belong to the NLR family and ABCG subfamily, which play an important role in the disease resistance mechanism of rice. Interestingly, the expression levels of five CNL genes and two ABCG genes on chromosome 9 were significantly increased in leaves after Y8 infection. At the same time, the expression level of one CNL gene on chromosome 10 was also significantly increased in stems. This finding reveals the differential regulation mechanism of rice disease resistance genes on different chromosomes. Further in-depth analysis showed that the regulatory network of related genes on chromosome 9 in leaves was complex, constituting an important gene pool of Yuanjiang common wild rice against bacterial blight. On the other hand, the resistance genes on chromosome 10 may not only be limited to resistance to bacterial blight, but they also play a key role in resistance to other stem diseases.

## Figures and Tables

**Table 1:** Functional annotation of proteins interacting with Os09g16458 and Os09g16449.

Interacting Proteins	Annotation	WoLF PSORT Prediction	CELLO v.2.5 Prediction
Os09g16458	ABC transporter domain-containing protein (ABCG subfamily genes protein)	Cytoplasmic	Cytoplasmic
Os09g16449	ABC transporter domain-containing protein (ABCG subfamily genes protein)	Plasma membrane	Plasma membrane
A0A0E0PK83	Abi domain-containing protein (Uncharacterized protein)	Nuclear, chloroplast	Plasma membrane
A0A0E0Q7S4	Uncharacterized protein (Uncharacterized protein)	Nuclear	Nuclear
A0A0E0Q7S6	Abi domain-containing protein (Uncharacterized protein)	Chloroplast, cytoplasmic	Plasma membrane
A0A0E0MWH1	DHquinase_I domain-containing protein (Uncharacterized protein)	Chloroplast, cytoplasmic	Plasma membrane, chloroplast
A0A0E0PGV6	HECT domain-containing protein (E3 ubiquitin-protein ligases)	Nuclear	Nuclear
A0A0E0PNN1	HECT domain-containing protein (E3 ubiquitin-protein ligases)	Cytoplasmic, nuclear	Nuclear, plasma membrane
A0A0E0PT33	Belongs to the EPSP synthase family, herbicide glyphosate-resistant protein	Chloroplast	Chloroplast
A0A0E0QKN4	COX15-CtaA domain-containing protein (Uncharacterized protein)	Chloroplast, plasma membrane	Plasma membrane
A0A0E0QUG4	DHQ synthase domain-containing protein	Chloroplast	Chloroplast
A0A0E0RI74	DHquinase_I domain-containing protein (Uncharacterized protein)	Chloroplast, mitochondrion	Mitochondrion

**Table 2:** Functional annotation of proteins interacting with CNL.

Preferred Name	Annotation	WoLF PSORT Prediction	CELLO v.2.5 Prediction
Os09g14010	Belongs to the disease resistance CC-NBS-LRR family	Chloroplast, nuclear	Nuclear
Os09g09750	Belongs to the disease resistance CC-NBS-LRR family	Chloroplast, nuclear	Cytoplasmic, nuclear
Os09g11020	Belongs to the disease resistance CC-NBS-LRR family	Cytoplasmic, chloroplast	Nuclear, cytoplasmic
Os09g34150	Belongs to the disease resistance CC-NBS-LRR family	Cytoplasmic	Cytoplasmic
Os09g34160	Belongs to the disease resistance CC-NBS-LRR family	Nuclear, cytoplasmic	Cytoplasmic, nuclear
A0A0E0MSI9	Belongs to the protein kinase superfamily	Cytoplasmic	Mitochondria, cytoplasmic
A0A0E0N210	F-box domain-containing protein	Chloroplast, nuclear, cytoplasmic	Plasma membrane, cytoplasmic
A0A0E0R8D7	AAA domain-containing protein belongs to the disease resistance NB-LRR family	Cytoplasmic	Cytoplasmic
A0A0E0RHG7	No functional domain belongs to the uncharacterized protein	Cytoplasmic	Cytoplasmic
A0A0E0P1Q4	Belongs to the protein kinase superfamily	Cytoplasmic	Cytoplasmic
A0A0E0Q8N6	Rx_N domain-containing protein, belongs to the uncharacterized protein	Cytoplasmic	Cytoplasmic
A0A0E0R6U5	Rx_N domain-containing protein, belongs to the uncharacterized protein	Cytoplasmic	Mitochondrial, nuclear
A0A0E0PEV5	DUF247 domain-containing protein belongs to the uncharacterized protein	Cytoplasmic, nuclear	Nuclear, cytoplasmic
A0A0E0QLH0	RING-type domain-containing protein	Nuclear	Nuclear
A0A0E0MY16	PWWP domain-containing protein	Nuclear	Nuclear
A0A0E0QUH2	DUF247 domain-containing protein belongs to the uncharacterized protein	Cytoplasmic, chloroplast, Nuclear	Plasma membrane, nuclear
A0A0E0PIN9	DUF247 domain-containing protein belongs to the uncharacterized protein	Plasma membrane	PlasmaMembrane
A0A0E0P9R8	F-box domain-containing protein	Chloroplast, mitochondria	Plasma membrane, nuclear
A0A0E0NF96	AvrRpt-cleavage domain-containing protein can be found in the RPM1 disease resistance	Vacuole, extracellular	Nuclear, extracellular

## Data Availability

The original contributions presented in the study are included in the article/Supplementary Materials, further inquiries can be directed to the corresponding author.

## Supplementary Materials

The following supporting information can be downloaded at: https://www.mdpi.com/article/10.3390/genes15070924/s1, Table S1: Fluorescence quantitative PCR primers designed based on 33 genes.
