# Supplementary material for: Examination of the Expression Profile of Resistance Genes in Yuanjiang Common Wild Rice (Oryza rufipogon)
Source: Genes (Basel). 2024 Jul 16;15(7):924. doi: 10.3390/genes15070924 (PMC11275508; doi:10.3390/genes15070924)
Supplement: Supplementary file 1 [file genes-15-00924-s001.zip › genes-3080867-supplementary.pdf]

Table S1. Fluorescence quantitative PCR primers designed based on 33 genes

Primer name	Forward primer（5′-3′）	Reverse primer（5′-3′）	Tm value
9-09490	TCCAGGTTGCAGGTTGATCC	AGCCAGCCTGAAAGGTCATC	60℃
9-09750	GGATCACACAGCGGCTGATA	ATTACCCGTCCACCTCCCAT	60℃
9-10054	TGTTTGTTGGAGAGCTGGCA	TTTCACTGCAACCGCAACAG	60℃
9-11020	ATGCGACAGTCTCACTTGTGT	CAAAGCAGGGAGGTTCGAGA	60℃
9-14010	CTGGAGACCTTTGAGCCAACA	TATGGCTGTGCGCATCATTG	60℃
9-14060	AATAATGTGTCGCATCGGCG	CCCTCCTTGCTTGCAGTTTTC	60℃
9-14100	GGTCATTCTTCGCACATCGC	AATTGGTCGATGCCACCTGT	63℃
9-15840	ATCAAGTCCCTGGCTTACGA	TCCGAAGAGCATCCATTACCTT	63℃
9-16000	GTCAACCCGACCCTCTCCC	GTAGAGCGCCCCCTTGATG	63℃
9-16330	GAAAAAGAGAGTAACCACAGGTAA T	AACAATGTGGATGGTCTGCC	60℃
9-16458	CTCGTGATCATGTGCTTGAGT	CCATGGTAAAACTCACTTCCTGC	60℃
9-16449	GTTGAGGCGCACGATCTTTT	CCAACAAACTTTACCTGTCCCA	60℃
9-16380	ACTCACCTCCAAACAGGGC	TGTGAGGCTTTACGATCCCAC	60℃
9-20020	CCTGACAAACACAACGGCGA	GAGCCCATGTAGAACCTCACT	60℃
9-20030	GGAACAGCGGTGTCCCTTC	CCACCAATGCCAGATATCGCT	60℃
9-20040	GAGATGGTGCCCCGACAATG	CGGAATTGACGACAGCAAGC	63℃
9-30220	GGCTTGTGGGACTTGAAGGA	AGGCTCCTCAGCTGGGATAA	63℃
9-30230	GGTCAACACAAGGGGTGTCT	AAAACCCAACCTGACCCTCC	63℃
9-34160	TTCGGTACCTCGAGACGCTA	AGTTGACGCCAGCAAGAAGA	60℃
9-34150	CGGATGGAACTGCCAAACTG	CACTGAAGTGGCCTTGTTCTT	63℃
10-03100	CTCAGCTCAAACTTGCTGGC	CCGGAGAAGGGGATGTAGGA	63℃
10-04090	GCACGCCTATCTCTTGTGTG	AGCAGCCCATGATGTGGTTTA	63℃
10-04342	CTGGGCAAGCTCAGTACCTT	CTTGCATGTTGCTCAGCTCC	63℃
10-04674	CTGGGCAAGCTCAGTACCT	GCTTGCATGTTGCTCAGCTC	63℃
10-07400	CAGTTGCCAGAAGCAGGACT	CAGTCAACGAGCCAATCCCT	63℃
10-07978	AGCTCTTAGCAAGGCACGAG	TTGAATCGACAAACGGCGTG	63℃
10-10360	CAGAGGCTTGGTGACCTTGT	AGCATTGCATCTGGCTCAGT	63℃
10-21400	GTAGGCTCCTCTTTGTGCGT	TGTTCCAGGCTTGGGTTGTT	63℃
10-22290	CTTCGTCTACGACCAGCTCA	GTCGTGTATCCTAACAGCCCC	63℃
10-22300	GTGACGGCCATCGTATGAGA	TGACAGGAACTCGAGAAGCG	63℃
10-25487	ATGTGCTATGGGGGTTTCCG	TCCTAAGCTGCGAAACAGGG	63℃
10-33440	CCTCGAATTCAGATTGTCCCCA	GGCGAGATCGGCTTCACTTC	63℃
10-36270	ATTGGACAATCTCTGCGGGG	GGAAGCCAAATGTGCCTGTG	63℃
Actin	CAGGCCGTCCTCTCTCTGTA	AAGGATAGCATGGGGGAGAG	60℃or63℃
